# Promising Antioxidant Activity of *Erythrina* Genus: An Alternative Treatment for Inflammatory Pain?

**DOI:** 10.3390/ijms22010248

**Published:** 2020-12-29

**Authors:** Tania Jiménez-Cabrera, Mirandeli Bautista, Claudia Velázquez-González, Osmar Antonio Jaramillo-Morales, José Antonio Guerrero-Solano, Thania Alejandra Urrutia-Hernández, Minarda De la O-Arciniega

**Affiliations:** 1 Institute of Health Sciences, Autonomous University of the State of Hidalgo, San Agustin Tlaxiaca 55679, Hidalgo, Mexico; tania_jimenez@uaeh.edu.mx (T.J.-C.); claudiav@uaeh.edu.mx (C.V.-G.); gsnutricional@gmail.com (J.A.G.-S.); thania_urrutia9356@uaeh.edu.mx (T.A.U.-H.); 2 Nursing and Obstetrics Department, Life Sciences Division, Campus Irapuato-Salamanca, Ex Hacienda El Copal, Km. 9 Carretera Irapuato-Silao, A.P 311, Irapuato 36500, Guanajuato, Mexico; oa.jaramillo@ugto.mx

**Keywords:** *Erythrina*, antioxidant, inflammatory pain, prenylated flavonoids

## Abstract

The negative impact that oxidative stress has on health is currently known. The complex mechanism of free radicals initiates a series of chain reactions that contribute to the evolution or development of different degenerative disorders. Likewise, these disorders are usually accompanied by inflammatory processes and, therefore, pain. In this sense, reactive oxygen species (ROS) have been shown to promote the nociceptive process, but effective treatment of pain and inflammation still represents a challenge. Over time, it has been learned that there is no single way to relieve pain, and as long as there are no other alternatives, the trend will continue to apply multidisciplinary management, such as promote the traditional use of the *Erythrina* genus to manage pain and inflammation. In this sense, the *Erythrina* genus produces a wide range of secondary metabolites, including flavanones, isoflavones, isoflavones, and pterocarpans; these compounds are characterized by their antioxidant activity. Phenolic compounds have demonstrated their ability to suppress pro-oxidants and inhibit inflammatory signaling pathways such as MAPK, AP1, and NFκB. Although there is preclinical evidence supporting its use, the pharmacological effect mechanisms are not entirely clear. Nowadays, there is a fast advancement in knowledge of the disciplines related to drug discovery, but most of nature’s medicinal potential has not yet been harnessed. This review analyzes the decisive role that the *Erythrina* genus could play in managing inflammatory pain mediated by its compounds and its uses as an antioxidant.

## 1. Molecular Origin of Inflammatory Pain

**Figure 1:**
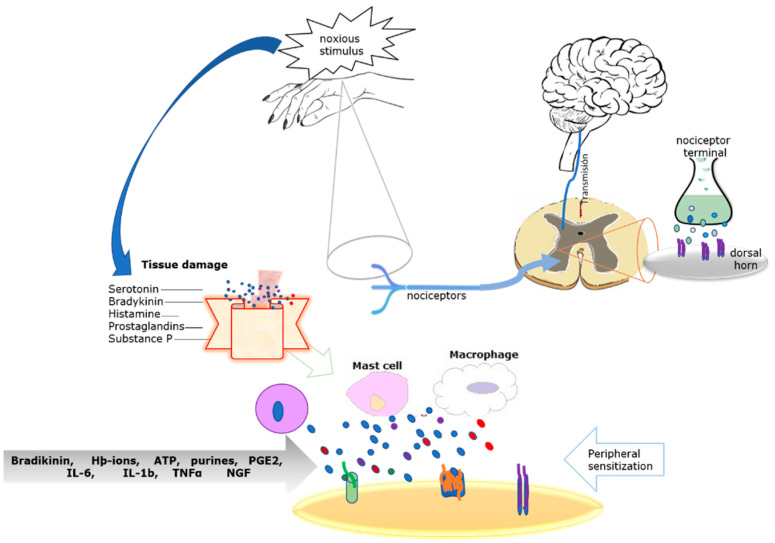
Diagram of the process of pain and peripheral sensitization leading to inflammatory pain.

Pain is traditionally defined as a complex sensory and emotional experience associated with actual or potential tissue damage or described in terms of such damage (International Association for the Study of Pain) [1,2,3]. It is a complex process that involves neuronal signaling pathways between the peripheral nervous system (PNS) and the central nervous system (CNS) [1,3]. The transduction of noxious stimuli (those that actually or potentially damage tissues) is carried out by a nociceptor, creating an electrophysiological neuronal signal encoded in the form of an action potential that is transmitted to the CNS. The acute injury is associated with a first, well-localized pain sensation transduced and transmitted by nociceptors. Although pain is one of the body’s most important adaptation and protection mechanisms, the degree of tissue damage leads to the release of inflammatory mediators that bind to its receptors, triggering an enzymatic cascade [1]. Thus, inflammatory pain is generated by an increase in sensitivity due to the cellular response associated with tissue damage, promoting the influx of activated cells such as macrophages, lymphocytes, and mast cells that release inflammatory mediators such as bradykinin, Hþ ions, ATP, purines, prostaglandin E2, leukotrienes, cytokines, nerve growth factor (NGF), sympathetic amines, and oxidative stress products present in the membrane of nociceptors [4] (Figure 1). NGF released by activated macrophages acts directly on peptidergic C fibers that express the TrkA receptor, a key component of peripheral sensitization. Macrophages release cytokines such as interleukin-6 (IL-6), IL-1b, tumor necrosis factor α (TNFα) which in turn contribute to peripheral sensitization through increased local production of proalgesic agents such as bradykinin, prostaglandins and increased release of NGF [1]. Prostaglandins are synthesized by consecutive reactions initiated by the phospholipase A2 enzyme that causes the release of arachidonic acid (AA) from cell membranes. Cyclooxygenase-2 (COX2) metabolizes arachidonic acid to prostaglandin G2 (PGG2) and prostaglandin H2 (PGH2) which is ultimately converted to PGE2 by prostaglandin E synthase (PTGES) [5]. PGE2 acts on all four E-prostanoid (EP) receptor subtypes (EP1-4). In peripheral tissue, PGE2 modulates pain sensitivity by sensitizing primary afferents. Sensitizes ion channels involved in pain, namely transient receptor potential vanilloid 1 channel, tetrodotoxin-sensitive Na+ channels, and purinergic P2X3 channels, also enhances the release of neurotransmitters. So, it is a crucial lipid mediator of inflammatory responses that causes pain hypersensitivity [6].

The pathological effects of ROS, IL-1β, TNFα, and IL-6 are due to the activation of various pro-inflammatory signaling pathways including the three (ERK, JNK, and p38) mitogen-activated protein kinases (MAPK), NFκB, AP1, and JAK/STAT [7]. There is evidence that mitochondrial dysfunction induced by oxidative and nitrosative stress leads to peripheral and central sensitization. Mammalian nerves are especially susceptible to free radicals, both oxygen (ROS) and nitrogen (RNS), due to the high content of phospholipids and axonal mitochondria; in addition to having a weak antioxidant system and other hand, some studies on antioxidant supplementation in animal models show that hydroxyl radicals (OH), superoxide (O_2_^−^), and nitric oxide (ON) may have a role in peripheral sensitization due to a deleterious effect on lipids and nucleic acids, protein carbonylation, and therefore the involvement of organelles and antioxidant enzymes [8]. Furthermore, ROS have also been shown to act as signaling molecules in a wide variety of cellular processes, including proliferation and survival (MAP kinases and PI3 kinase) and the regulation of antioxidant genes (Ref-1 and Nrf-2) [7]. PI3K catalyzes the synthesis of the second messenger phosphatidylinositol 3,4,5-triphosphate (PIP3) from phosphatidylinositol 4,5-bisphosphate (PIP2), wherein the membrane-bound PIP3 serves as a signaling molecule to recruit proteins containing the pleckstrin homology (PH) domain. These PH domain proteins, such as the phosphoinositide-dependent protein kinase (PDK) and protein kinase B (AKT) serine/threonine kinases are thus activated and mediate further downstream signaling events. Both PI3K and MAPK are similarly regulated by ROS at the oxidative interface, where protein phosphatases are directly oxidized by ROS, resulting in sustained activation of signaling pathways [7]. According to the International Association for the Study of Pain (IASP), although comprehensive epidemiological data are not worldwide available, almost 50% of adults suffer from more than one type of pain [9]. Pain is associated with most of the diseases, however, particularly the skin, joints, and intestines are susceptible to the development of inflammatory pain, and its prevalence is increased [10]. Therefore, the effective management of pain and inflammation represents a challenge in clinical research, as the scientific discipline of pain management is a relatively new field of research [4].

Nowadays, the general population suffers different types of collateral damage, which leads to the need to find more effective drugs, with fewer side effects and greater accessibility, to eliminate the inflammatory process and the associated pain. Furthermore, nociceptive stimuli do not always respond to common analgesics or NSAIDs, so other therapies or therapeutic options [11] as natural products, are used. They represent a desirable approach for developing new drugs, particularly useful in patients with inflammatory pain [12] the antioxidant activity of natural products assumes a decisive role in the management of inflammation and accompanying pain [13]. Antioxidants already known, such as vitamin E, resveratrol, or quercetin have shown tables analgesic and anti-inflammatory properties due to the properties that their chemical structure confers on them [14,15]. Although today there is rapid growth and advancement in knowledge of the disciplines related to drug discovery, the medicinal potential of most of nature has not yet been harnessed [12].

The species of genus *Erythrina*, have a great variety of medicinal properties. It has been widely used in folk therapies due to their curare and hypnotic functions and their associated pharmacological effects, including sedatives, hypotensives, neuromuscular blockers, and central nervous system (CNS) depressants [16,17]. Besides, they have been also used to treat microbial infections [18], inflammation [19], amenorrhea, headache, eye problems, female sterility, liver disorders, asthma, and malaria diseases [20,21,22]. 

## 2. Ethnomedicinal Use of the Genus *Erythrina*

This genus is a member of the legume family (*Fabaceae*), subfamily *Papilionoideae.* It comprises at least 120 species most of which are trees and some perennials with large woody roots [23]. These species are collectively called “coral trees”, alluding to the flowers, characteristics of the genus, which are commonly bright red [24]. The place of origin of the genus *Erythrina* is not exactly known, but it is suggested that it was probably in South America, since most of the supposed “primitive” groups within the genus are found there. 70 species are recognized in the Neotropics, 38 in Africa and Madagascar, and 12 in Asia and Australia [23].

Although there is local ethnobotanical data on the use of the genus *Erythrina* to relieve pain and inflammation, few preclinical studies to evaluate the effect have been published. An even smaller amount describes the biological activity and therapeutic potential of the genus for this purpose. Furthermore, the relationship between antioxidant properties and anti-inflammatory effect has not been analyzed. Therefore, this review will focus on the ethnopharmacological analysis of the genus *Erythrina* and the decisive role that it could play in the management of inflammatory pain.

There are some papers that documented the ethnomedicinal use of different species from *Erythrina* genus in the treatment of pain and/or inflammation, such as: *E. abyssinica*, *E. caffra* [25,26,27,28,29,30] and *E. arborences* [31,32] are the most used species in traditional medicine. Table 1 shows a summary of the species studied the part of the tree used, the method of preparation and their ethnomedicinal use. The bark and leaves are the common part of the plant used for medicinal purposes. The decoction is the habitual form of preparation, the liquid obtained is ingested or applied externally on the affected area.

## 3. Preclinical Studies of Pain and Inflammation from the Genus *Erythrina*

**Figure 2:**
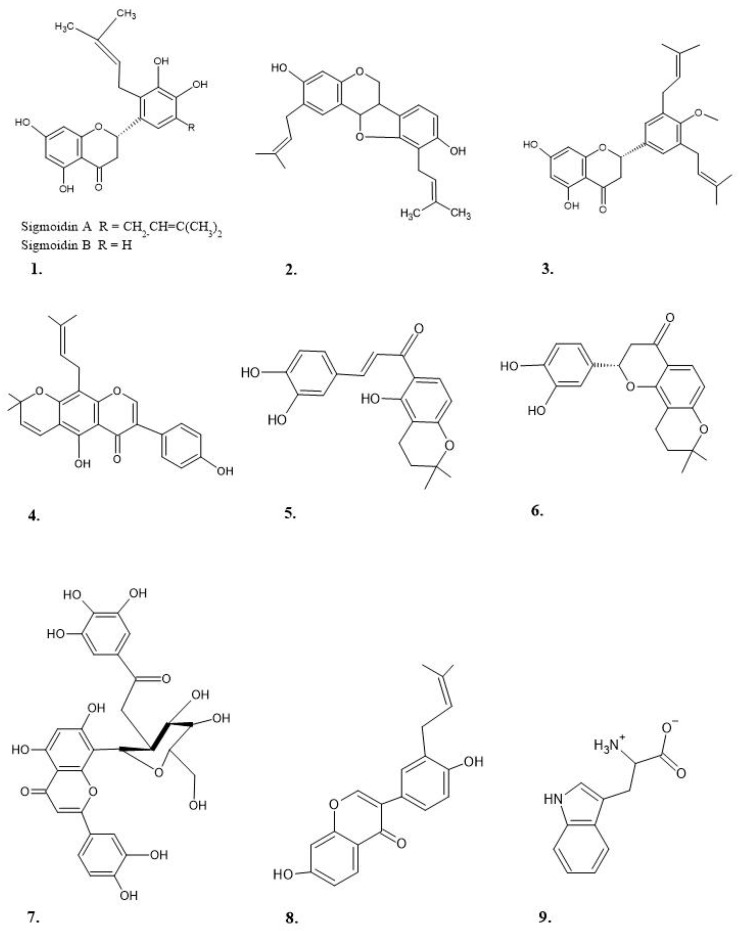
Main chemical structures are involved in the inhibition of the inflammatory process. Sigmoidin A and B (**1**), Erycristagallin (**2**), Abyssinone V-4′-methyl ether (**3**), Waragalone (**4**), Mildbenone (**5**), Mildbone (**6**), 2″-O-galloyl orientin (**7**), Neobavaisoflavone (**8**) and Hypaphorine (**9**). The chemical structures were created with the ACD/ChemSketch Freeware program.

Table 2 shows preclinical studies on the analgesic and anti-inflammatory effect of species of the genus *Erythrina* genus. *E. variegate* was the most studied species, both for its analgesic and anti-inflammatory effect [36,37,38,39,40], followed by *E. velutina* and *E. mulungu* [41,42,43]. Mostly, high polarity solvents (ethanol, water, and methanol) were used to obtain the extracts. This suggests that the biological activity demonstrated, in the various studies, was largely due to polar compounds. Mostly, high polarity solvents (ethanol, water, and methanol) were used to obtain the extracts. This suggests that the biological activity demonstrated in the various studies was largely due to polar compounds. Two flavanones (Sigmoidin A and B), a prenylated flavonoid (abyssinone V-4′-methyl ether), a prenylisoflavone (warangalone), and a pterocarpane (Erycristagallin) (Figure 2), demonstrated anti-inflammatory properties at doses of 300–600 mg/kg [44,45,46], all compounds demonstrated marked anti-inflammatory efficacy. The anti-inflammatory suggested mechanisms include the inhibition of prostaglandins and cyclooxygenases. The most widely used reference drugs in pain models were morphine and diclofenac. While for the inflammation models it was Indomethacin, diclofenac, and dexamethasone. Although in some cases, acetylsalicylic acid and Pentazocin were used. In general, all preclinical studies agree that each species studied is shown to have an analgesic and/or anti-inflammatory effect [36,40,41,46]. Antagonistic effects with histamine and/or serotonin were also mentioned [40,46]. Although their causes are not clarified, blocking of HRs and 5-HT receptors are related [47]. Likewise, the participation of antioxidant activity in the regulation of anti-inflammatory and analgesic processes through the inhibition of nitric oxide (NO) is highlighted [45,48,49]. According to the authors, the compounds involved in these mechanisms are mainly flavonoids. However, it is also mentioned that alkaloids erysotrine, erysotrine hypophorine, reduced the number of inflammatory cells in lung tissue, mainly eosinophils and lymphocytes. Possibly due to the decrease of IL-4 and IL-5, which stimulate the maturation of eosinophils in the bone marrow and recruit these cells to the tissues. In turn, this can impact the modulation of the synthesis and release of inflammatory mediators, such as prostaglandins, nitric oxide, and cytokines such as IL-1 and TNF α [19]. Docking studies shows that phaseollin of *Erythrina variegata* has the best fitness score against the COX-1 which is 56.64 and 59.63 for COX-2 enzyme [50,51,52,53,54,55,56,57]. However, is required to delve into the possible mechanisms of action, as well as the phytoconstituents and their relationship with the biological activity [58].

## 4. Radical Scavenging Activity in the Model In Vitro Systems

Various methods are used to investigate the antioxidant property of different samples. Can be classified so in vitro and in vivo antioxidant models [64]. The antioxidant activity of the extracts and/or compounds of *E. abyssinica*, *E. livingstoniana* and *E. mildbraedii* it proved in different studies [51,55,56,65,66]. In vitro radical scavenging assay (DPPH) was the most widely used in vivo test to determine the capacity of free radical scavenging (Table 3). In most studies, a similar and even higher activity was obtained than the positive controls (Trolox, BHA, ascorbic acid and quercetin) [58,65,66,67,68]. Reduction of Fe ions was also evaluated, an assay often used as an indicator of electron donation activity (FRAP). Additionally, the in vivo antioxidant activities of the enzymes SOD, CAT, and GSH were measured to evaluate the hepatoprotective potential of *Erythrina indica*, *senegalensis*, and × *neillii*, in rats. Where the activities of antioxidant enzymes were restored (*p* < 0.05) [69]. Inhibition of lipoxygenase and xanthine oxidase, enzymes that participate in the production of reactive oxygen species and pro-inflammatory agents were other tests used [55,56,70]. Likewise, the decrease in lipid peroxidation (TBARS) and the inhibition of NO were used to evaluate the antioxidant properties [52,53,71,72]. Among the compounds responsible for these activities, Eryvarin H, Abyssinone V, mildbone, mildbenone, 7,3′-dihydroxy-4′-methoxy-5′-(3 methylbut-2-enyl)flavanone, erylivingstone H, 7,3′,4′-trihydroxyflavanone, trans-3,4,2′,4′-tetrahydroxychalcone, Eryvarin J and erycrisagallin [46,55,65,68,73,74,75]. Flexible molecular docking on heme oxygenase, an important stress protein that is involved in cellular protection, antioxidant and anti-inflammatory activities, showed with 2″-O-galloyl orientin forming four binding interactions with residues, Arg 136 (two interactions), Met34 and Gly139 [39]. On the other hand, it has been reported that the compound abyssinone V increases oxidative stress and reduces stress resistance in the Caenorhabditis elegans model [76]. However, many antioxidant compounds are also evaluated for their cytotoxic activity that promotes apoptosis favoring a pro-oxidant environment. This is highly dependent on the used concentrations of the compound. However, studies are required to help clarify this activity. 

As mentioned earlier an increase in free radicals exacerbates the inflammatory response. Likewise, it has been observed that supplementation with antioxidants in animal models can decrease peripheral sensitization caused by ROS. For which it is suggested that compounds with antioxidant activity of the genus *Erythrina* may play an important role in the modulation of inflammatory pain.

Although medicinal plants often have many different uses within and between cultures, much remains to be investigated about the species of this genus in terms of their potential in treating pain and inflammation. 

## 5. Molecular Mechanisms of Anti-Inflammatory Activity of the Genus *Erythrina*

Medicinal plants and their secondary metabolites, such as polyphenols and alkaloids, have long been considered valuable sources of natural remedies for the treatment of human diseases [86]. At least 25% of modern medicines come directly or indirectly from plant origin [12,87]. However, even today with the rapid growth and advance in the knowledge of disciplines related to drug discovery, it may be that most of nature’s medicinal potential has yet to be tapped [12].

Such is the case of the genus *Erythrina*, which is made up of a great variety of species and characterized by producing a wide range of secondary metabolites. At least 370 flavonoid compounds have been isolated, including flavones, flavonols, flavanones, chalcones, isoflavans, isoflav-3-enos, neoflav-3-ene, isoflavanones, isoflavones, pterocarpans, coumestanes, arylcoumarins, coumaryl benin chromones that include flavoflavones, isoflavones, isoflavanones, pterocarpanes and pre-C-erythrine alkaloids and approximately 143 alkaloids distributed mainly in seeds, stem, bark, leaves and flowers [37,88]. 

Pain is always associated with the region where the inflammation is located and can become chronic if the inflammation is not relieved quickly [1]. Several mediators are involved in the inflammatory pathway, prostaglandins, leukotrienes, cytokines, platelet-activating factor, and chemokines [89]. Likewise, during the inflammatory process reactive oxygen and nitrogen species are also produced along with different proteases that can cause tissue damage, fibrosis, and cell proliferation, which can contribute to the chronicity of inflammation [90]. Behind these processes, there is a complex signaling network between the immune system and injured tissue [91]. The deepening of the knowledge of inflammatory pain will allow to optimize and accelerate the development of innovative therapeutic targets of natural origin [92]

The *Erythrina* alkaloids have been of interest for their structural characteristics and their variety of biological activities [93,94]. The dihydro-β-erythroidine alkaloid was used to characterize the nicotinic acetylcholine receptors (nAChRs) [94], a preferential antagonist of the α4β2 nicotinic receptor subunit that acts as a competitive inhibitor like erisothrin, erisopine, erisodine [59]. nAChRs are involved in several central nervous system (CNS) disease states, including depression, schizophrenia, attention deficit hyperactivity disorder, Alzheimer’s and Parkinson’s diseases, substance abuse, and pain [95,96]. On the other hand, it has been suggested that nAChRs may represent viable targets for new analgesics [96,97]. These receptors are widely distributed throughout the CNS, expressing themselves in neurons and non-neuronal cells [98]. In recent years, α7 nAChRs in macrophages has been shown to regulate inflammation, activating the “cholinergic anti-inflammatory pathway” [98,99,100]. There is accumulating evidence suggesting that α7 nAChR agonists and modulators are promising targets for the treatment of chronic inflammatory pain [101] The treatment with *E. mulungu* extract significantly reduced the levels of pro-inflammatory cytokines, as well as the infiltration of inflammatory cells in lung tissue. The main compounds identified in the extract were erisothrin, erisothrin-N-oxide and hypaphorine (Figure 1). The cholinergic anti-inflammatory pathway allows the suppression of inflammation, it was characterized by its effects on the release of cytokines by macrophages. This pathway allows the suppression of inflammation by vagal efferents depending on α7 nAChRs [99]. Hypaphorine, an anti-inflammatory compound [102] has been isolated from many *Erythrina* species and other plant species [98,102,103,104]. A study carried out by Aswad 2017 determined that hypaphorine is one of the molecules that is considered as a potential candidate for an anti-inflammatory drug. Hypophorine present in *Vaccaria segetalis* demonstrated downregulation of cyclooxygenase-2 (COX-2) and inducible nitric oxide synthase (iNOS). Furthermore, it delayed LPS-induced phosphorylation of ERK, and immunofluorescence staining revealed that *Vaccaria* hypophorine eliminated nuclear translocation of NFκB in LPS-treated RAW 264.7 cells [102]. During inflammation, the action of α7 nAChR is associated with the entry of calcium and the interruption of the stimulation of nuclear factor κB (NF κB) [105]. It is possible that *Eryhrina* hypaphorine regulates the inflammatory process through α7 nAChRs, activating a cholinergic anti-inflammatory pathway.

Several studies have shown that the anti-inflammatory activity of some polyphenols depends on their ability to suppress pro-inflammatory signaling pathways such as MAPK, AP1, and NFκB, and in turn, this ability is associated with the ability to restore a suitable redox environment [106,107]. Among the members of the MAPK cascades, apoptosis signal-regulated kinase 1 (ASK1) is an upstream MAPKKK that regulates the JNK and p38 MAPK pathways. ASK1 is activated under various stress conditions including oxidative stress. ASK1-deficient mouse embryonic fibroblasts were decreased JNK and p38 MAPK activation. ROS-activated ASK1 mediates p38 signaling pathway leading to nonapoptotic outcomes that probably favor the increase of pro-inflammatory cells [7]. Flavonoids are molecules of interest due to their biological effects observed *in vitro*. Their potential utility as antibiotic agents have been validated [18] anti-allergy [108] anti-diarrhea [109] antiulcer [110] anti-inflammatory [49,90] and analgesics [60]. At first, it was considered that the main mechanism of action of antioxidant compounds lays in their ability to scavenge radicals directly. Although the mechanisms that participate in these processes have not been studied in depth. The possible mechanisms may be related to effects on intracellular and intercellular signaling pathways, regulation of nuclear transcription factors, fat metabolism, and modulation in the synthesis of inflammatory [111]. Since the inhibition of pro-inflammatory enzymes, such as cyclooxygenase-2 (COX-2), lipoxygenase (LOX) and inducible nitric oxide synthase (iNOS) has been demonstrated. Pretreatment of primary chondrocytes and cartilage explants with Imperatorin, a plant secondary metabolite belonging to the family of furanocoumarins, suppressed the production of iNOS and NO, blocking IL-1β-induced phosphorylation of the ERK-MAPK/AP1 signaling pathway [106]. Inhibition of protein kinases such as phosphoinositol kinase, protein kinase C, phosphatidylinositol kinase has been documented, tyrosine kinase, or cyclin-dependent kinase-4. As well as the activation of phase II detoxifying pathways through the activation of factor 2 related to erythroid nuclear factor 2 (Nrf2). Additionally. Additionally, several flavonoids can decrease the expression of different pro-inflammatory cytokines/chemokines, including TNFα, IL-1β, IL-6, IL-8, and the monocyte chemoattractant protein-1 (MCP-1), in different types of cells, such as RAW macrophages, Jurkat T cells, and peripheral blood mononuclear cells [90,112]. Phenolic compounds isolated and characterized from *E. neillii* exhibited the highest antioxidant activity, principally 2″-O-galloyl orientin, followed by 2″-O-galloyl vitexin. Additionally. Additionally, flexible molecular docking on heme oxygenase (HO-1), an important stress protein that is involved in cellular protection, antioxidant and anti-inflammatory activities, justified the antioxidant activity of the isolated compounds [58]. One of the pathways implicated in the control of inflammation Nrf2 that controls the expression of antioxidant response element-regulated antioxidant and cyto-protective genes, such as NAD(P)H: quinone oxidoreductase 1 (NQO1), γ-glutamyl cysteine synthetase catalytic subunit (GCLC), and heme oxygenase (HO)-1 [113]. From what can be suggested, 2″-O-galloyl orientin, maybe a potential activator of Nrf2 and therefore play a fundamental role in the treatment of inflammatory pain.

The hydroalcoholic extracts of *E. indica* and *E. suberosa,* have shown percentages of inhibition of nitric oxide 21.5% to 89%. Given the polarity of the solvent used for the extraction, the participation of phenolic compounds in the reduction of nitric oxide was suggested [54,69]. The production of NO and prostaglandins is regulated by iNOS and COX-2. iNOS is distinguished by generating more NO than the constituent members, it is involved in the development and maintenance of central and peripheral sensitization in inflammatory and neuropathic pain. NO can act as a neurotransmitter that affects spinal nociceptive processing in various pain models. COX-2 in macrophages increases in an oxidizing environment and, in turn, increases inflammatory responses [114]. So too, the ethanol and ethyl acetate bark extracts of *E. caffra*, *E. latissima,* and *E. lysistemon* exhibited an important cyclooxygenase inhibiting activity. *E. caffra* and *E. lysistemon* displayed inhibition of more than 90% for both the 50 and 500 mg/mL doses, this suggested the presence of potent compounds in the bark, as flavonoids. *E. caffra* is one of *Erythrina* species most frequently used by traditional healers to relieve inflammation [48]. On the other hand, the administration of ethanolic extract of leaves from *E. neillii* at 100, 250, and 500 mg/kg in the methotrexate-intoxicated rats significantly mitigated lipid peroxidation activity, with a significant decrease of malondialdehyde (MDA) in the hepatic tissue, and a significant increase of GSH and SOD activity, in a dose-dependent manner. Additionally. Additionally, a significant decrease in the hepatic tissue content of TNF-α was demonstrated [71]. Likewise, the GSH, SOD, and CAT content significantly increased (*p* < 0.05 to *p* < 0.001) in the groups treated with the methanol extract of *E. indica* leaves [52]. All this compared with silymarin a potent antioxidant. It was suggested that flavones such Liquiritin, derived from plant licorice, increased SOD, CAT, GSH-PX enzymatic activity through activating the Nrf2/keap1 pathway and attenuation the ERK1/2/NF-κB pathway [115]. Although it has been documented that the pathological effects of ROS on inflammation are due to the activation of several pro-inflammatory signaling pathways such as mitogen-activated protein kinases (MAPK) [105], recently shown that some prenylated flavonoids induce the expression of HO-1 activating Nrf2 through the p38 MAPK pathway [113,116,117].

NF-κB is a transcription factor that plays an important role in the transcription of genes, which are involved in immune and inflammatory responses. It was recently observed that Toll-like receptor 7 contributes to neuropathic pain by activating NF-κB in primary sensory neurons and subsequently induced the release of inflammatory mediators in immune cells [118]. NF-κB is released from a complex with I-κB and migrates to the nucleus where it binds to the κB enhancer element and induces transcription of its target genes, such as COX-2, iNOS, TNF-α, IL-1β, and IL -6, chemokines and adhesion molecules [27,114]. Treatment with an *E. speciosa* methanol extract (ESLE) induced a significant reduction in the immunoexpression of NF-κB, COX-2, iNOS, and the pro-inflammatory marker, TNF-α in doses of 50–100 mg/kg. Additionally. Additionally, it increased the levels of GSH and catalase. This study concludes that ESLE exerted a gastroprotective effect through the synergistic anti-inflammatory and antioxidant activity of its various compounds, such as flavonoids (orientin, isoorientin, vitexin, isovitexin, and luteolin), alkaloids (hypaphorine), and saponins [88]. It has been seen that an oxidative environment produces a positive regulation of cytokines and macrophages, increasing the inflammatory response mediated by COX-2. The cytokines released can act directly on the primary afferent sensory neurons increasing the permeability of sodium (Na^+^) sensitizing the capsaicin receptors (TRPV1). These effects facilitate peripheral sensitization. Likewise, the oxidative environment can activate brain regions that transmit pain signals from other brain regions, which are responsible for nociceptive processing, such as the rostral ventrolateral medulla (RMV) [119]. The methanolic extract from the leaves of *E. senegalensis* has recently been seen, it showed a protective effect against oxidative stress in SC-1 fibroblasts and THP-1 macrophages. Observing the highest antioxidant activity against free radicals (IC50 = 44.86 μg/mL [ABTS]; 291.1 μg/mL [DPPH]) and intracellular ROS induced by 2,2′-Azobis (2-amidinopropane) dihydrochloride (AAPH) in macrophages. This effect was comparable to that of the positive control, Trolox [70]. The presence of compounds such as kaempferol, rutin, and rotenone is suggested as possible, responsible for the effect, however, confirmation of the finding is required [85]. However, neobavaisoflavone (Figure 1), an isoflavone previously isolated from the root of *E. senegalensis* and *E. excelsa* [120] and found in *Psoralea corylifolia* L., has been shown to have significant anti-inflammatory and antioxidant activity [121,122].

The sources that precede the generation of reactive oxygen species (ROS) can be both endogenous and exogenous. B (XO) located on the outer surface of the plasma membrane and in the cytoplasm. It catalyzes the oxidation of hypoxanthine to xanthine and then to uric acid. The production of xanthine and XO is greatly increased during ischemia, accompanied by the loss of antioxidant enzymes. O_2_^−^ is an electron acceptor and XO cofactor, thus generating O_2_^−^ and H_2_O_2_, one of the main ROS in ischemia, causing damage to ischemic cells and different tissues [123]. Gout, a common and complex form of arthritis that can affect anyone, is characterized by sudden and intense attacks of pain, swelling, redness, and tenderness in the joints. Its treatment consists of increasing uric acid excretion or reducing uric acid production [21]. Xanthine oxidase inhibitors (XOI) are particularly useful, as they have minor side effects compared to uricosuric and anti-inflammatory agents (NSAIDs and corticosteroids). However, allopurinol, the only clinically used XOI, also causes side effects such as rash, low blood cells, and hypersensitivity syndrome. In this context, it has been shown that the chloroform fraction of *E. stricta* leaves inhibits xanthine oxidase in a concentration-dependent manner. Although in vitro inhibition was moderate compared to allopurinol, at higher doses (21.2 y 100 µg/mL) XO was significantly inhibited. It was suggested that the presence of phenolic and flavonoid content in the extract contributed to the inhibition of XO [70]. For their part, [53], concluded that the methanolic extract of the stem bark of *E. indica* has a strong activity in the inhibition of XO. Additionally, it has been shown that the methanol extract of the root has an important antioxidant activity and a strong inhibition of NO [54]. Some of the compounds isolated in this species include isoflavones such as genistein, wighteone, alpinumisoflavone, dimethylalpinumisoflavone, 8-prenilerithrinin C, and erisenegalensein E, from stem bark [55], compounds that may be responsible for the effect.

Mildbone and mildbenone, flavanone and chalcone from *E. mildbraedii* exhibited significant antioxidant and moderate LOX inhibition activities [55,56] participates in the eicosanoid cascade during the inflammatory response, using arachidonic acid as a substrate, for the synthesis of leukotrienes (LT) and other oxidized lipids intermediates [124]. So, these compounds acted as an anti-inflammatory. At least three inhibitory mechanisms of LOX have been recognized, among these, some compounds act on an essential iron atom at the site, affecting its oxidation state (redox inhibitors) and binding directly to the iron atom (chelators), affecting the cycle catalytic. This non-selective antioxidant mechanism [124] may be associated with the antioxidant capacity of *E. mildbraedii* compounds and be responsible for the observed effect. The authors suggest that the high antioxidant activity of mildbone is mainly related to the second bond dissociation enthalpy (BDE) of a second hydrogen atom transfer from i-OH phenoxyl radical to the free radical [55]. The BDEs of the respective phenol bonds correlate with the antioxidant effect of these compounds. It was found that phenols with low BDE values lead to clearly higher stabilizations [125]. Mildbone with lower IC50 and BDE had greater antioxidant effects than mildbenone [55]. These values are generated by group Electrondonators (EDG). Hydroxy groups (–OH) represent EDG and therefore lead to lower BDE values. This makes it clear that high antioxidant activity is mainly related to the number and positions of OH groups located in ring B [125,126]

Prenylated flavonoids are a subclass of flavonoids, combining a flavonoid skeleton with a lipophilic prenyl side chain. They are compounds of low abundance in nature. Prenylation provides these compounds with improved biological activity. Increases the lipophilicity of flavonoids, providing a greater affinity for biological membranes and a better interaction with target proteins [127,128]. It was found that pterocarpan erycristagallin decreased the edema induced by phospholipase A2 by 51% in the first 30 min, however, the effect disappeared 60 min after application. This suggests that the effect may be an indirect inhibition of the enzyme. In rat peritoneal leukocytes, the application of erycristagallin inhibited the production of leukotriene B4 (metabolite 5-lipoxygenase). This compound showed purifying properties, inhibiting the stable free radical DPPH by 96% at a concentration of 50 AM, while the reference drug quercetin produced inhibition of 92% at the same dose. Previous studies have suggested that different antioxidant agents and free radical scavengers can reduce 5-lipoxygenase activity through a mechanism that interferes with divalent ions involved in catabolism of arachidonic acid [129,130]. Besides, some prenylated flavonoids have been shown to have the ability to inhibit COX or LOX activity, depending on substitution patterns. Sigmoidins A and B, prenylated flavanones from *E. sigmoidea*, contain a catechol group in ring B and a 2′5-diphenyl group (Sigmoidin A) or a 2′-prenyl group (sigmoidin B). At a concentration of 100 nm, sigmoidin A clearly inhibited leukotriene B4 production in rat polymorphonuclear leukocytes by 100%, while the same dose of sigmoidin B only reduced production by 44%. In PLA_2_-induced paw edema, sigmoidin B showed a clear inhibitory effect against the induction of edema (59% at 60 min) while sigmoidin A had only a mild effect at 30 min. Since sigmoidin B did not affect arachidonate metabolism, it was suggested that it affected histamine release [49]. Another prenylated flavonoid, the prenylisoflavone warangalone isolated from the bark of *E. addisoniae*, is a potent inhibitor of protein kinase A and showed marked anti-inflammatory efficacy on phospholipase A2-induced paw edema and 12-induced ear edema. O-tetradecanoylphorbol 13-acetate in mice [46]. Prenylated flavonoids have been reported to act as anti-inflammatory, through five mechanisms that include antioxidant and radical scavenging activities; regulation of the activities of cells related to inflammation; modulation of the activities of the enzymes of arachidonic acid metabolism (phospholipase A_2_, cyclooxygenase, lipoxygenase) and nitric oxide synthase; modulation of the production of other pro-inflammatory molecules and the modulation of the expression of pro-inflammatory genes [128,131].

At least 370 flavonoid compounds have been identified, in the genus *Erythrina.* Among them, several prenylated flavonoids [114,132,133,134,135,136,137] that due to their characteristic structures, have a better interaction with the target molecules. However, the role of these compounds in inflammatory pain has not been explored. Prenylated flavonoids have a very restricted application due to their lower abundance in the environment. However, the lack of preclinical studies on these compounds present in the species of the genus *Erythrina* indicates that most of the medicinal potential of this genus has not yet been exploited.

## 6. Methods

### 6.1. Search Strategy

An organized search for the ethnomedicinal use of the genus *Erythrina* in the treatment of pain and inflammation was conducted, as well as the preclinical studies performed.

The search was carried out systematically using MeSH (Medical Subject Headings) terms and “keywords”. First, we define the related MeSH terms: “Anti-inflammatory agents”, “analgesics/therapeutic use” “analgesics [Pharmacological action]”, “pain management”, “ethnobotany” “medicinal plant”, “ethnopharmacology” “antioxidant activity” and “flavonoids”, then each term was combined with *Erythrina*. Subsequently, keywords such as: “pain relief” “antinociceptive effect” “anti-inflammatory effect”, “ethnopharmacological studies” and “antioxidants” were combined with “*Erythrina*”.

### 6.2. Inclusion Criteria

All articles published from 2000 to May 2020, found in the scientific information sources ScienceDirect, Medline (Pubmed) and Springer link, were considered.

A selection of titles was made, from which the abstracts were read and those that met the necessary characteristics were retrieved. The following criteria were included for the selection of documents.

In the case of the ethnomedicinal reports, the documents that expose the use of the different parts of species of the genus *Erythrina* in the relief of pain and/or inflammation were selected.

In preclinical studies, studies that describe species of the genus *Eythrina* and models to evaluate analgesic and/or anti-inflammatory activity (in vivo and in vitro), including dose, reference drug, possible mechanisms of action, as well as the main metabolites. associated with the effect were selected.

Regarding antioxidant activity, articles were selected that mentioned the type of test used, the species studied and the type of extract and/or compounds evaluated.

Items that did not meet the requirements were discarded.

## 7. Conclusions

Inflammatory pain, as a pathological phenomenon, has been established throughout history as a public health problem. There are several pro-inflammatory mediators involved in the complex web of the process. However, the data presented here show that the phytoconstituents of the genus *Erythrina* have the potential capacity to modulate different therapeutic targets and those effects could be associated with their antioxidant properties. Several compounds with antioxidant, analgesic, and anti-inflammatory effects have been identified. Among them, several prenylated flavonoids that, due to their characteristic structures, have a better interaction with the target molecules. However, the lack of preclinical studies on these compounds and the species of the genus *Erythrina* indicates that most of the medicinal potential of this genus has not yet been explored. Likewise, it is necessary to delve into the molecular mechanisms involved in both effects.

## Figures and Tables

**Table 1:** Species of the genus *Erythrina* used in traditional medicine for the relief of pain or inflammation.

Species	Part Used	Folk Use	Administration	Reference
*E. abyssinica*	Bark	Inflammation, backache, pain and cramps lower belly	Decoction, external use, extract drunk and boiled in milk.	[25,27,33]
*E. arborences*	Branch, seed and leaves	Bone fracture and back pain	Paste/fomentation, decoction oral, juice of leaves	[31,32]
*E. caffra*	Bark, leaves and roots	Sprains, aches	Decoction oral, eardrops and plaster	[26,28,29]
*E. caffra* *E. lysistemon*	Stem bark and leaves	Toothache and earache	Oral infusion	[30]
*E. edulis*	Bark	Headache	Aqueous infusion drink	[34]
*E. humeana*	Bark	Spraians	Decoction, external use	[26]
*E. senegalensis*	Bark	Inflammation and Backache	Decoction, external use, massage with ointment	[22]
*E. variegata*	Leaves and bark	Fever, body ache, chronic bronchitis and otalgia	Decoction, oral	[35]

**Table 2:** Preclinical studies on the analgesic and anti-inflammatory effect of species of the genus Erythrina.

Species	Extract	Part	Model	Reference
*E. addisoniae*	EtOAc and MeOH	Stem bark	Inhibition of leukotriene B4 production from rat polymorphonuclear leukocytes. Cyclooxygenase-1 (COX-1) activity from human platelets. PLA2 induced paw oedema in mouse. TPA-induced mouse ear oedema.	[46]
*E. lysistemon* *E. latissima* *E. humeana* *E. zeyheri*	Ethanol and ethyl acetate	Leaves and bark	Cyclooxygenase-1 inhibition	[48]
*E. indica*	MeOH	Leaves	Carrageenan-induced hind paw edema	[59]
*E. droogmansina*	Ethyl acetate and MeOH	Root bark	Carrageenan-induced hind paw edema Ear edema induced by xylene Cotton pellet-induced granuloma	[45]
*E. crista-galli*	EtOH (70%) Dichlorometane MeOH	Aerial parts	Writhing test, Formalin test, Hot-plate	[60]
*E. mildbraedii*	Ethyl acetate	Root bark	Carrageenan-induced hind paw edema PLA_2_ induced paw oedema in mouse TPA-induced mouse ear oedema	[57]
*E. mulungu*	EtOH and EtOH 30%	Flowers and stem bark	Ovalbumin (OVA)-induced asthma in mice Dextran induced paw edema	[19,41,61]
*E. senegalensis*	Aqueous and EtOH (70%)	Bark and roots	Writhing test Egg albumin induced paw edema in rats. Hot-plate	[62,63]
*E. sigmoidea*	Chloroform	Bark	Inhibition of leukotriene B4 production from rat polymorphonuclear leukocytes. Cyclooxygenase-1 (COX-1) activity from human platelets. PLA2 induced paw oedema in mouse. TPA-induced mouse ear oedema.	[49]
*E. variegata*	MeOH, EtOH (95%), EtOH and Aqueous.	Leaves and bark	Writhing test, Tail-flik Carrageenan-induced hind paw edema Cotton pellet induced granuloma Hot plate HRBC membrane stabilization	[36,39,40]
*E. velutina*	EtOH (30%) and Aqueous	Stem bark and leaves	Writhing test, Formalin test, Hot-plate Carrageenan-induced hind paw edema	[41,62]

**Table 3:** Antioxidant activity of species from genus Erythrina.

Species	Part	Identified Extract or Compounds	Model	Reference
*E. abyssinica*	Stem bark and root	Erycristagallin (4), 3-hydroxy-9-methoxy-10-(3,3-dimethylallyl) pterocarpene and 7,3′,4′-trihydroxy-5′-prenylflavanone (Abyssinone VII)	DPPH	[50,51]
*E. burttii*	Root bark	Burttinol-A and burttinol-C, and the 2-arylbenzofuran derivative burttinol-D	DPPH	[77]
*E. crista-galli*	Bark	Alkaloids, erythraline, erythrinine and hypaphorine	Inhibitory activity on LPS-induced nitric oxide (NO)	[72]
*E. droogmansiana*	Root bark	Genistein, 3-(3′,4′-methelenedioxyphenyl)-2,3-epoxypropanol, asperphenamate, Erydroogmansin B, vogelin C, Isolupalbigenin and erypostyrene	DPPH and FRAP	[78,79]
*E. edulis*	Seeds	Protein concentrate from the seed flour	ABTS, DPPH and ORAC	[80]
*E. indica*	Leaves and stem bark	Methanol extract	DPPH) and Nitric oxide scavenging assay	[52,53]
*E. livingstoniana*	Stem bark and twing	7,3′-dihydroxy-4′-methoxy-5′-(3-methylbut-2-enyl) flavanone, 7, 3′,4′-trihydroxyflavanone and trans-3,4,2′,4′-tetrahydroxychalcone	DPPH	[65,81]
*E. variegata*	Leaves and bark	Methanolic extract and crude polysaccharides	DPPH, FRAP and TEAC	[67,82]
*E. mildbraedi*	Roots and bark	Flavanone (mildbone), chalcone (mildbenone) and Pterocarpene (Erycristagallin)	DPPH	[55,56,83]
*E. senegalensis*	Stem bark and leaves	Hydroalcoholic extract fraction 3 (polyphenols and flavonoids) and Methanol extract	DPPH, ABTS and FRAP	[84,85]
*E. sigmoidea*	Stem bark	Methanol extract; Flavanones, Sigmoidin A and Sigmoidin B	DPPH	[49,67]
*E. stricta*	Leaves	Hydromethanolic extract was	In vitro xanthine oxidase inhibitory activity All	[70]
*E. suberosa*	Flowers	Methanol extract	DPPH and Nitric oxide scavenging assay	[69]
*E. vogelii*	Leaves	Ethanol extract	DPPH	[67]
*E. neillii*	Leaves	Methanol total extract and its fractions	ORAC	[58]

## Abbreviations

ROS	Reactive oxygen species
MAPK	Mitogen-Activated Protein Kinase
AP1	Activator protein 1
NF-kB	Nuclear factor kappa-light-chain-enhancer of activated B cells
ERK	Extracellular signal-regulated kinases
JNK	Jun N-Terminal Kinase
JAK	Janus kinases
STAT	Signal Transducer and Activator of Transcription
IASP	International Association for the Study of pain
NSAIDs	Nonsteroidal anti-inflammatory drugs
CNS	Central Nervous System
NO	Nitric Oxide
DPPH	2,2-diphenyl-1-picrylhydrazyl
BHA	Butylated hydroxyanisole
NAChRs	Nicotinic Acetylcholine Receptors
COX-2	Cyclooxygenase 2
iNOs	Nitric oxide synthase, inducible
LOX	Lipoxygenases
Nrf2	Nuclear factor erythroid 2-related factor 2
TNFα	Tumor Necrosis Factor alpha
IL-1β	Interleukin-1-beta
IL-6	Interleukin-6
IL-8	Interleukin-8
HO-1	Heme Oxygenase-1
GCLC	γ-glutamyl cysteine synthetase catalytic subunit
NAD(P)H	Nicotinamide adenine dinucleotide phosphate
GSH	Glutathione
SOD	Superoxide dismutase
CAT	Catalase
ELSE	*E. speciosa* methanol extract
TRPV1	Transient receptor potential cation channel subfamily V member 1
RMV	Rostral ventrolateral medulla
XO	Xanthine oxidase
XOI	Xanthine oxidase inhibitor
BDE	Second bond dissociation enthalpy
EDG	Group Electrondonators

## References

[1] Hudspith M.J. (2016). Anatomy, physiology and pharmacology of pain. Anaesth. Intensive Care Med..

[2] Bentley N., Awad A.J., Patil P.G. (2018). Physiology and Pathophysiology of Chronic Pain. Neuromodulation.

[3] Ellison D.L. (2017). Physiology of Pain. Crit. Care Nurs. Clin. N. Am..

[4] Tompkins D.A., Hobelmann J.G., Compton P. (2017). Providing chronic pain management in the “Fifth Vital Sign” Era: Historical and treatment perspectives on a modern-day medical dilemma. Drug Alcohol Depend..

[5] Kakavandi N., Rezaee S., Hosseini-Fard S.R., Ghasempour G., Khosravi M., Shabani M., Najafi M. (2021). Prostaglandin E2 (PGE2) synthesis pathway is involved in coronary artery stenosis and restenosis. Gene.

[6] Kanda H., Kobayashi K., Yamanaka H., Okubo M., Dai Y., Noguchi K. (2021). Localization of prostaglandin E2 synthases and E-prostanoid receptors in the spinal cord in a rat model of neuropathic pain. Brain Res..

[7] Gupta R.K., Patel A.K., Shah N., Chaudhary A.K., Jha U.K., Yadav U.C., Gupta P.K., Pakuwal U. (2014). Oxidative stress and antioxidants in disease and cancer: A review. Asian Pac. J. Cancer Prev..

[8] Carrasco C., Naziroglu M., Rodríguez A.B., Pariente J.A. (2018). Neuropathic pain: Delving into the oxidative origin and the possible implication of transient receptor potential channels. Front. Physiol..

[9] Dahlhamer J., Lucas J., Zelaya C., Nahin R., Mackey S., DeBar L., Kerns R., von Korff M., Porter L., Helmick C. (2018). Prevalence of Chronic Pain and High-Impact Chronic Pain Among Adults—United States, 2016. Mmwr. Morb. Mortal. Wkly. Rep..

[10] Muley M.M., Krustev E., Mcdougall J.J. (2016). Preclinical Assessment of Inflammatory Pain. CNS Neurosci. Ther..

[11] Vargas J.R.N., Pinzón J.E.C. (2012). The labyrinth of pain and the need for fostering basic research: El laberinto del dolor y la necesidad de impulsar la investigación básica. Colomb. J. Anesthesiol..

[12] Robinson M.M., Zhang X. (2011). The World Medicines Situation 2011 Traditional Medicines: Global Situation, Issues and Challenges.

[13] Benvenuti S., Bortolotti E., Maggini R. (2016). Antioxidant power, anthocyanin content and organoleptic performance of edible flowers. Sci. Hortic..

[14] Li S.H., Li L., Yang R.N., Liang S.D. (2020). Compounds of traditional Chinese medicine and neuropathic pain. Chin. J. Nat. Med..

[15] Lee F.H., Raja S.N. (2011). Complementary and alternative medicine in chronic pain. Pain.

[16] Faggion S.A., Cunha A.O.S., Fachim H.A., Gavin A.S., dos Santos W.F., Pereira A.M.S., Beleboni R.O. (2011). Anticonvulsant profile of the alkaloids (+)-erythravine and (+)-11-α-hydroxy-erythravine isolated from the flowers of Erythrina mulungu Mart ex Benth (Leguminosae-Papilionaceae). Epilepsy Behav..

[17] Santos Rosa D., Faggion S.A., Gavin A.S., Anderson de Souza M., Fachim H.A., Ferreira dos Santos W., Soares Pereira A.M., Cunha A.O.S., Beleboni R.O. (2012). Erysothrine, an alkaloid extracted from flowers of Erythrina mulungu Mart. ex Benth: Evaluating its anticonvulsant and anxiolytic potential. Epilepsy Behav..

[18] Akter K., Barnes E.C., Loa-Kum-Cheung W.L., Yin P., Kichu M., Brophy J.J., Barrow R.A., Imchen I., Vemulpad S.R., Jamie J.F. (2016). Antimicrobial and antioxidant activity and chemical characterisation of Erythrina stricta Roxb. (Fabaceae). J. Ethnopharmacol..

[19] Amorim J., Borges M., de Carvalho Borges M., Fabro A.T., Contini S.H.T., Valdevite M., Pereira A.M.S., Carmona F. (2019). The ethanolic extract from Erythrina mulungu Benth. flowers attenuates allergic airway inflammation and hyperresponsiveness in a murine model of asthma. J. Ethnopharmacol..

[20] Mukungu N., Abuga K., Okalebo F., Ingwela R., Mwangi J. (2016). Medicinal plants used for management of malaria among the Luhya community of Kakamega East sub-County, Kenya. J. Ethnopharmacol..

[21] Bodofsky S., Merriman T.R., Thomas T.J., Schlesinger N. (2020). Advances in our understanding of gout as an auto-inflammatory disease. Semin. Arthritis Rheum..

[22] Togola A., Austarheim I., Theïs A., Diallo D., Paulsen B. (2008). Ethnopharmacological uses of Erythrina senegalensis: A comparison of three areas in Mali, and a link between traditional knowledge and modern biological science. J. Ethnobiol. Ethnomedicine.

[23] de Luca A., Sibilio G., de Luca P., del Guacchio E. (2018). DNA barcoding to confirm the morphological identification of the coral trees (Erythrina spp., fabaceae) in the ancient gardens of Naples (Campania, Italy). Plants.

[24] Kumar A., Lingadurai S., Jain A., Barman N. (2010). Erythrina variegata Linn: A review on morphology, phytochemistry, and pharmacological aspects. Pharmacogn. Rev..

[25] Schlage C., Mabula C., Mahunnah R.L.A., Heinrich M. (2000). Medicinal plants of the Washambaa (Tanzania): Documentation and ethnopharmacological evaluation. Plant Biol..

[26] Corrigan B.M., van Wyk B.E., Geldenhuys C.J., Jardine J.M. (2011). Ethnobotanical plant uses in the KwaNibela Peninsula, St Lucia, South Africa. S. Afr. J. Bot..

[27] Pompermaier L., Marzocco S., Adesso S., Monizi M., Schwaiger S., Neinhuis C., Stuppner H., Lautenschläger T. (2018). Medicinal plants of northern Angola and their anti-inflammatory properties. J. Ethnopharmacol..

[28] Dzoyem J.P., McGaw L.J., Eloff J.N. (2014). In vitro antibacterial, antioxidant and cytotoxic activity of acetone leaf extracts of nine under-investigated Fabaceae tree species leads to potentially useful extracts in animal health and productivity. BMC Complement. Altern. Med..

[29] Farag M.A., Mekky H., El-Masry S. (2016). Metabolomics driven analysis of Erythrina lysistemon cell suspension culture in response to methyl jasmonate elicitation Erythrina lysistemon cell culture metabolomics. J. Adv. Res..

[30] Mhlongo L.S., van Wyk B.E. (2019). Zulu medicinal ethnobotany: New records from the Amandawe area of KwaZulu-Natal, South Africa. S. Afr. J. Bot..

[31] Wangchuk P., Yeshi K., Jamphel K. (2017). Pharmacological, ethnopharmacological, and botanical evaluation of subtropical medicinal plants of Lower Kheng region in Bhutan. Integr. Med. Res..

[32] Lee S., Xiao C., Pei S. (2008). Ethnobotanical survey of medicinal plants at periodic markets of Honghe Prefecture in Yunnan Province, SW China. J. Ethnopharmacol..

[33] Maroyi A. (2011). An ethnobotanical survey of medicinal plants used by the people in Nhema communal area, Zimbabwe. J. Ethnopharmacol..

[34] Tene V., Malagón O., Finzi P.V., Vidari G., Armijos C., Zaragoza T. (2007). An ethnobotanical survey of medicinal plants used in Loja and Zamora-Chinchipe, Ecuador. J. Ethnopharmacol..

[35] Kamble M.Y., Mane S.S., Murugan C., Jaisankar I. (2018). Diversity of Ethno-Medicinal Plants of Tropical Islands-with Special Reference to Andaman and Nicobar Islands.

[36] Bhagyasri Y., Nagalatha G., Reddy N.V., Subramanian N.S. (2017). Analgesic and anti-inflammatory activity of leaf extracts of Erythrina variegate. Indo Am. J. Pharm. Res..

[37] Fahmy N.M., Al-Sayed E., El-Shazly M., Singab A.N. (2018). Comprehensive review on flavonoids biological activities of Erythrina plant species. Ind. Crop. Prod..

[38] Haque R., Ali M.S., Saha A., Alimuzzaman M. (2006). Analgesic Activity of Methanolic Extract of the Leaf of Erythrina variegata. Dhaka Univ. J. Pharm. Sci..

[39] Nasir Uddin M.M., Emran T.B., Mahib M., Dash R. (2014). Molecular docking and analgesic studies of Erythrina variegata’s derived phytochemicals with COX enzymes. Bioinformation.

[40] Krishna Raju Mantena V.R., Tejaswini G. (2015). Anti inflammatory activity of Erythrina Variegata. Int. J. Pharm. Pharm. Sci..

[41] Vasconcelos S.M.M., Rebouças Oliveira G., Mohana De Carvalho M., Rodrigues A.C.P., Rocha Silveira E., Maria França Fonteles M., Florenço Sousa F.C., Barros Viana G.S. (2003). Antinociceptive activities of the hydroalcoholic extracts from Erythrina velutina and Erythrina mulungu in mice. Biol. Pharm. Bull..

[42] Vasconcelos S.M.M., Macedo D.S., de Melo C.T.v., Monteiro A.P., Cunha G.M.A., Sousa F.C.F., Viana G.S.B., Rodrigues A.C.P., Silveira E.R. (2004). Central activity of hydroalcoholic extracts from Erythrina velutina and Erythrina mulungu in mice. J. Pharm. Pharmacol..

[43] Marchioro M., Blank M.D.F.A., Mourão R.H.V., Antoniolli Â.R. (2005). Anti-nociceptive activity of the aqueous extract of Erythrina velutina leaves. Fitoterapia.

[44] Cui L., Thuong P.T., Lee H.S., Ndinteh D.T., Mbafor J.T., Fomum Z.T., Oh W.K. (2008). Flavanones from the stem bark of Erythrina abyssinica. Bioorg. Med. Chem..

[45] Sokeng S.D., Talla E., Jeweldai V., Yaya A.J.G., Koube J., Dongmo F., Goulimé M., Mbafor J.T. (2013). Anti-inflammatory effect of Abyssinone V-4′-methyl ether on acute and chronic inflammation models. Hygeia J. Drugs Med..

[46] Talla E., Njamen D., Mbafor J.T., Fomum Z.T., Kamanyi A., Mbanya J.C., Giner R.M., Recio M.C., Máñez S., Ríos J.L. (2003). Warangalone, the isoflavonoid anti-inflammatory principle of Erythrina addisoniae stem bark. J. Nat. Prod..

[47] Branco A.C.C.C., Yoshikawa F.S.Y., Pietrobon A.J., Sato M.N. (2018). Role of Histamine in Modulating the Immune Response and Inflammation. Mediat. Inflamm..

[48] Njamen D., Mbafor J.T., Fomum Z.T., Kamanyi A., Mbanya J.C., Recio M.C., Giner R.M., Máñez S., Ríos J.L. (2004). Anti-inflammatory activities of two flavanones, sigmoidin A and sigmoidin B, from Erythrina sigmoidea. Planta Med..

[49] Pillay C.C.N., Jäger A.K., Mulholland D.A., van Staden J. (2001). Cyclooxygenase inhibiting and anti-bacterial activities of South African Erythrina species. J. Ethnopharmacol..

[50] Yenesew A., Twinomuhwezi H., Kiremire B.T., Mbugua M.N., Gitu P.M., Heydenreich M., Peter M.G. (2009). 8-Methoxyneorautenol and radical scavenging flavonoids from Erythrina abyssinica. Bull. Chem. Soc. Ethiop..

[51] MacHumi F., Bojase-Moleta G., Mapitse R., Masesane I., Majinda R.R.T. (2006). Radical scavenging-flavonoids from Erythrina abyssinica. Nat. Prod. Commun..

[52] Mujahid M., Hussain T., Siddiqui H.H., Hussain A. (2017). Evaluation of hepatoprotective potential of Erythrina indica leaves against antitubercular drugs induced hepatotoxicity in experimental rats. J. Ayurveda Integr. Med..

[53] Sowndhararajan K., Joseph J.M., Rajendrakumaran D. (2012). In vitro xanthine oxidase inhibitory activity of methanol extracts of Erythrina indica Lam. leaves and stem bark. Asian Pac. J. Trop. Biomed..

[54] Sre P.R.R., Sheila T., Murugesan K. (2012). Phytochemical screening and “in-vitro” anti-oxidant activity of methanolic root extract of Erythrina indica. Asian Pac. J. Trop. Biomed..

[55] Anouar E.H. (2016). Antioxidant activity of mildbone and mildbenone secondary metabolites of Erythrina mildbraedii Harms: A theoretical approach. Comput. Theor. Chem..

[56] Ali M.S., Ali M.I., Ahmed G., Afza N., Lateef M., Iqbal L., Waffo A.F.K., Ahmed Z. (2012). Potent antioxidant and lipoxygenase inhibitory flavanone and chalcone from Erythrina mildbraedii harms (Fabaceae) of cameroon. Z. Fur Nat.-Sect. B J. Chem. Sci..

[57] Njamen D., Talla E., Mbafor J.T., Fomum Z.T., Kamanyi A., Mbanya J.C., Cerdá-Nicolás M., Giner R.M., Recio M.C., Ríos J.L. (2003). Anti-inflammatory activity of erycristagallin, a pterocarpene from Erythrina mildbraedii. Eur. J. Pharmacol..

[58] Gabr S.K., Bakr R.O., Mostafa E.S., El-Fishawy A.M., El-Alfy T.S. (2019). Antioxidant activity and molecular docking study of Erythrina × neillii polyphenolics. S. Afr. J. Bot..

[59] Verma S.M., Prakash J., Sah V.K. (2005). Phyto-Pharmacognostical Investigation and Evaluation of Anti-Inflammatory and Sedative Hypnotic Activity of the Leaves of Erythrina Indica Lam. Anc. Sci. Life.

[60] Fischer L.G.O., Leitão R., Etcheverry S.R., de Campos-Buzzi F., Vãzquez A.A., Heinzen H.A., Filho V.C. (2007). Analgesic properties of extracts and fractions from Erythrina crista-galli (Fabaceae) leaves. Nat. Prod. Res..

[61] Vasconcelos S.M.M., Sales G.T.M., Lima N., Lobato R.d.F.G., Macêdo D.S., Barbosa-Filho J.M., Leal L.K.A.M., Fonteles M.M.F., Sousa F.C.F., Oliveira J.L. (2011). Anti-inflammatory activities of the hydroalcoholic extracts from Erythrina velutina and E. Mulungu in mice. Braz. J. Pharmacogn..

[62] Musa A., Nazifi A.B., Usman A.I., Kassim A. (2016). Evaluation of analgesic and behavioural effects of ethanol root bark extract of Erythrina senegalensis DC ( Fabaceae ). UoN Protal.

[63] Saidu K., Onah J., Orisadipe A., Olusola A., Wambebe C., Gamaniel K. (2000). Antiplasmodial, analgesic, and anti-inflammatory activities of the aqueous extract of the stem bark of Erythrina senegalensis. J. Ethnopharmacol..

[64] Alam M.N., Bristi N.J., Rafiquzzaman M. (2013). Review on in vivo and in vitro methods evaluation of antioxidant activity. Saudi Pharm. J..

[65] Bedane K.G., Kusari S., Masesane I.B., Spiteller M., Majinda R.R.T. (2016). Flavanones of Erythrina livingstoniana with antioxidant properties. Fitoterapia.

[66] Bedane K.G., Kusari S., Eckelmann D., Masesane I.B., Spiteller M., Majinda R.R.T. (2015). Erylivingstone A-C with antioxidant and antibacterial activities from Erythrina livingstoniana. Fitoterapia.

[67] Tauseef S., Ali M.S., Ahmed A., Ali M.I., Ahmed Z., Sherwani S.K., Ahmed G., Onocha P.A., Joseph N., Francois A. (2013). In vitro Antioxidant activity analysis of five medicinally important plants. J. Pharmacogn. Phytochem..

[68] Zhang C., Zhou Y., Sun Z., Feng J., Wang Y. (2014). Polysaccharides extraction from Erythirna variegata, chemical characterization and its antioxidant activity. Int. J. Biol. Macromol..

[69] Janbaz K.H., Nisar U., Ashraf M., Qadir M.I. (2012). Spasmolytic, bronchodilatory and antioxidant activities of Erythrina superosa Roxb. Acta Pol. Pharm.-Drug Res..

[70] Umamaheswari M., Asokkumar K., Sivashanmugam A.T., Remyaraju A., Subhadradevi V., Ravi T.K. (2009). In vitro xanthine oxidase inhibitory activity of the fractions of Erythrina stricta Roxb. J. Ethnopharmacol..

[71] Bakr R.O., Fayed M.A.A., Fayez A.M., Gabr S.K., El-Fishawy A.M., Taha S.E.A. (2019). Hepatoprotective activity of Erythrina × neillii leaf extract and characterization of its phytoconstituents. Phytomedicine.

[72] Ozawa M., Kawamata S., Etoh T., Hayashi M., Komiyama K., Kishida A., Kuroda C., Ohsaki A. (2010). Structures of new erythrinan alkaloids and nitric oxide production inhibitors from Erythrina crista-galli. Chem. Pharm. Bull..

[73] Gurmessa G.T., Kusari S., Laatsch H., Bojase G., Tatolo G., Masesane I.B., Spiteller M., Majinda R.R.T. (2018). Chemical constituents of root and stem bark of Erythrina brucei. Phytochem. Lett..

[74] Yenesew A., Irungu B., Derese S., Midiwo J.O., Heydenreich M., Peter M.G. (2003). Two prenylated flavonoids from the stem bark of Erythrina burttii. Phytochemistry.

[75] Yenesew A., Midiwo J.O., Heydenreich M., Schanzenbach D., Peter M.G. (2000). Two isoflavanones from the stem bark of Erythrina sacleuxii. Phytochemistry.

[76] Koch K., Schulz G., Döring W., Büchter C., Havermann S., Mutiso P.C., Passreiter C., Wätjen W. (2019). Abyssinone V, a prenylated flavonoid isolated from the stem bark of Erythrina melanacantha increases oxidative stress and decreases stress resistance in Caenorhabditis elegans. J. Pharm. Pharmacol..

[77] Yenesew A., Akala H.M., Twinomuhwezi H., Chepkirui C., Irungu B.N., Eyase F.L., Kamatenesi-Mugisha M., Kiremire B.T., Johnson J.D., Waters N.C. (2012). The antiplasmodial and radical scavenging activities of flavonoids of Erythrina burttii. Acta Trop..

[78] Bedane K.G., Kusari S., Bullach A., Masesane I.B., Mihigo S.O., Spiteller M., Majinda R.R.T. (2017). Chemical constituents of the root bark of Erythrina droogmansiana. Phytochem. Lett..

[79] Yaya A.J.G., Feumba R.D., Emmanuel T., Tchinda A.T., Fredérich M., Oben J., Mbafor J.T. (2014). Antioxidant activity of compounds isolated from the root woods of Erythrina droogmansiana. Int. J. Pharm. Sci. Drug Res..

[80] Intiquilla A., Jimenez K., Zavaleta A.I. (2016). Erythrina edulis (Pajuro) Seed Protein: A New Source of Antioxidant Peptides. Natural Product Communications.

[81] Bedane K.G., Masesane I.B., Majinda R.R.T. (2016). New isoflavans from the root bark of Erythrina livingstoniana. Phytochem. Lett..

[82] Alam M.K., Rana Z.H., Islam S.N., Akhtaruzzaman M. (2020). Comparative assessment of nutritional composition, polyphenol profile, antidiabetic and antioxidative properties of selected edible wild plant species of Bangladesh. Food Chem..

[83] Jang J.P., Na M.K., Thuong P.T., Njamen D., Mbafor J.T., Fomum Z.T., Woo E.R., Oh W.K. (2008). Prenylated flavonoids with PTP1B inhibitory activity from the root bark of Erythrina mildbraedii. Chem. Pharm. Bull..

[84] Donfack J.H., Nico F.N., Ngameni B., Tchana A., Chuisseu P.D., Finzi P.V., Ngadjui B.T., Moundipa P.F. (2008). In vitro hepatoprotective and antioxidant activities of diprenylated isoflavonoids from Erythrina senegalensis (Fabaceae). Pharmacologyonline.

[85] Yahaya E.S., Cordier W., Steenkamp P.A., Steenkamp V. (2020). Protective effect of Erythrina senegalensis sequential extracts against oxidative stress in SC-1 fibroblasts and THP-1 macrophages. J. Pharm. Pharmacogn. Res..

[86] Leonti M., Stafford G.I., Cero M.D., Cabras S., Castellanos M.E., Casu L., Weckerle C.S. (2017). Reverse ethnopharmacology and drug discovery. J. Ethnopharmacol..

[87] Anywar G., Namukobe J. (2020). Factors Affecting the Choice for Plant-Based Products in Drug Discoveries.

[88] Fahmy N.M., Al-Sayed E., El-Shazly M., Nasser Singab A. (2020). Alkaloids of genus Erythrina: An updated review. Nat. Prod. Res..

[89] Feehan K.T., Gilroy D.W. (2019). Is Resolution the End of Inflammation?. Trends Mol. Med..

[90] Maleki S.J., Crespo J.F., Cabanillas B. (2019). Anti-inflammatory effects of flavonoids. Food Chem..

[91] Elgorashi E.E., McGaw L.J. (2019). African plants with in vitro anti-inflammatory activities: A review. S. Afr. J. Bot..

[92] Aswad M., Rayan M., Abu-Lafi S., Falah M., Raiyn J., Abdallah Z., Rayan A. (2018). Nature is the best source of anti-inflammatory drugs: Indexing natural products for their anti-inflammatory bioactivity. Inflamm. Res..

[93] Amaral P.d.A., Antunes A.R., Barlow J.W. (2019). Isolation of erythrinan alkaloids from the leaves and flowers of Erythrina speciosa. Rev. Bras. De Farmacogn..

[94] Wu J., Zhang B.J., Bao M.F., Cai X.H. (2019). A new erythrinan N-oxide alkaloid from Erythrina stricta. Nat. Prod. Res..

[95] Crestey F., Jensen A.A., Borch M., Andreasen J.T., Andersen J., Balle T., Kristensen J.L. (2013). Design, synthesis, and biological evaluation of Erythrina alkaloid analogues as neuronal nicotinic acetylcholine receptor antagonists. J. Med. Chem..

[96] Umana I.C., Daniele C.A., McGehee D.S. (2013). Neuronal nicotinic receptors as analgesic targets: It’s a winding road. Biochem. Pharmacol..

[97] Gao B.X., Hierl M., Clarkin K., Juan T., Nguyen H., Valk M.v.d., Deng H., Guo W., Lehto S.G., Matson D. (2010). Pharmacological effects of nonselective and subtype-selective nicotinic acetylcholine receptor agonists in animal models of persistent pain. Pain.

[98] Egea J., Buendia I., Parada E., Navarro E., León R., Lopez M.G. (2015). Anti-inflammatory role of microglial alpha7 nAChRs and its role in neuroprotection. Biochem. Pharmacol..

[99] Ben-David Y., Kagan S., Cohen Ben-Ami H., Rostami J., Mizrahi T., Kulkarni A.R., Thakur G.A., Vaknin-Dembinsky A., Healy L.M., Brenner T. (2020). RIC3, the cholinergic anti-inflammatory pathway, and neuroinflammation. Int. Immunopharmacol..

[100] Pohanka M. (2012). Alpha7 nicotinic acetylcholine receptor is a target in pharmacology and toxicology. Int. J. Mol. Sci..

[101] Setti-Perdigão P., Serrano M.A.R., Flausino O.A., Bolzani V.S., Guimarães M.Z.P., Castro N.G. (2013). Erythrina mulungu alkaloids are potent inhibitors of neuronal nicotinic receptor currents in mammalian cells. PLoS ONE.

[102] Sun H., Cai W., Wang X., Liu Y., Hou B., Zhu X., Qiu L. (2017). Vaccaria hypaphorine alleviates lipopolysaccharide-induced inflammation via inactivation of NFΚB and ERK pathways in Raw 264.7 cells. BMC Complement. Altern. Med..

[103] El-masry S., Hammoda H.M., Zaatout H., Abdel-kader M. (2010). Constituents of Erythrina caffra Stem Bark Grown in Egypt. Nat. Prod. Sci..

[104] Ozawa M., Honda K., Nakai I., Kishida A., Ohsaki A. (2008). Hypaphorine, an indole alkaloid from Erythrina velutina, induced sleep on normal mice. Bioorg. Med. Chem. Lett..

[105] Ansari M.Y., Ahmad N., Haqqi T.M. (2020). Oxidative stress and inflammation in osteoarthritis pathogenesis: Role of polyphenols. Biomed. Pharmacother..

[106] Ahmad N., Ansari M.Y., Bano S., Haqqi T.M. (2020). Imperatorin suppresses IL-1β-induced iNOS expression via inhibiting ERK-MAPK/AP1 signaling in primary human OA chondrocytes. Int. Immunopharmacol..

[107] Khan H., Sureda A., Belwal T., Çetinkaya S., Süntar İ., Tejada S., Devkota H.P., Ullah H., Aschner M. (2019). Polyphenols in the treatment of autoimmune diseases. Autoimmun. Rev..

[108] Lee D.H., Park J.K., Choi J., Jang H., Seol J.W. (2020). Anti-inflammatory effects of natural flavonoid diosmetin in IL-4 and LPS-induced macrophage activation and atopic dermatitis model. Int. Immunopharmacol..

[109] Afroz N., Ahsanul Hoq M., Jahan S., Mainul Islam M., Ahmed F., Shahid-Ud-Daula A.F.M., Hasanuzzaman M. (2020). Methanol soluble fraction of fruits of Annona muricata possesses significant antidiarrheal activities. Heliyon.

[110] Fahmy N.M., Al-Sayed E., Michel H.E., El-Shazly M., Singab A.N.B. (2020). Gastroprotective effects of Erythrina speciosa (Fabaceae) leaves cultivated in Egypt against ethanol-induced gastric ulcer in rats. J. Ethnopharmacol..

[111] Fraga C.G., Croft K.D., Kennedy D.O., Tomás-Barberán F.A. (2019). The effects of polyphenols and other bioactives on human health. Food Funct..

[112] Serafini M., Peluso I., Raguzzini A. (2010). Flavonoids as anti-inflammatory agents. Proc. Nutr. Soc..

[113] Tran P.L., Tran P.T., Tran H.N.K., Lee S., Kim O., Min B.S., Lee J.H. (2018). A prenylated flavonoid, 10-oxomornigrol F, exhibits anti-inflammatory effects by activating the Nrf2/heme oxygenase-1 pathway in macrophage cells. Int. Immunopharmacol..

[114] Nguyen T.H., Nachtergael A., Nguyen T.M., Cornet V., Duez P., Muller M., Ly Huong D.T., Kestemont P. (2020). Anti-inflammatory properties of the ethanol extract from Clerodendrum cyrtophyllum Turcz based on in vitro and in vivo studies. J. Ethnopharmacol..

[115] Li X., Qin X., Tian J., Gao X., Wu X., Du G., Zhou Y. (2020). Liquiritin protects PC12 cells from corticosterone-induced neurotoxicity via regulation of metabolic disorders, attenuation ERK1/2-NF-κB pathway, activation Nrf2-Keap1 pathway, and inhibition mitochondrial apoptosis pathway. Food Chem. Toxicol..

[116] Gandhi M., Nair S. (2020). New vistas in malignant mesothelioma: MicroRNA architecture and NRF2/MAPK signal transduction. Life Sci..

[117] Zhao J., Piao X., Wu Y., Liang S., Han F., Liang Q., Shao S., Zhao D. (2020). Cepharanthine attenuates cerebral ischemia/reperfusion injury by reducing NLRP3 inflammasome-induced inflammation and oxidative stress via inhibiting 12/15-LOX signaling. Biomed. Pharmacother..

[118] He L., Han G., Wu S., Du S., Zhang Y., Liu W., Jiang B., Zhang L., Xia S., Jia S. (2020). Toll-like receptor 7 contributes to neuropathic pain by activating NF-κB in primary sensory neurons. Brainbehaviorand Immun..

[119] Nashed M.G., Balenko M.D., Singh G. (2014). Cancer-induced oxidative stress and pain. Curr. Pain Headache Rep..

[120] Kuete V., Sandjo L.P., Kwamou G.M.N., Wiench B., Nkengfack A.E., Efferth T. (2014). Activity of three cytotoxic isoflavonoids from Erythrina excelsa and Erythrina senegalensis (neobavaisoflavone, sigmoidin H and isoneorautenol) toward multi-factorial drug resistant cancer cells. Phytomedicine.

[121] Xu J., Li M., Yao Z., Zhang Y., Li S., Hu L., Qin Z., Gonzalez F.J., Yao X. (2018). In vitrometabolic mapping of neobavaisoflavone in human cytochromes P450 and UDP-glucuronosyltransferase enzymes by ultra high-performance liquid chromatography coupled with quadrupole time-of-flight tandem mass spectrometry. J. Pharm. Biomed. Anal..

[122] Ye H., He X., Feng X. (2020). Developing neobavaisoflavone nanoemulsion suppresses lung cancer progression by regulating tumor microenvironment. Biomed. Pharmacother..

[123] Srdí-Rají T., Koní Ristí A. (2015). Antioxidants: Role on Health and Prevention. Encycl. Food Health.

[124] Wood I., Trostchansky A., Rubbo H. (2020). Structural considerations on lipoxygenase function, inhibition and crosstalk with nitric oxide pathways. Biochimie.

[125] Kerkel F., Brock D., Touraud D., Kunz W. (2021). Stabilisation of biofuels with hydrophilic, natural antioxidants solubilised by glycerol derivatives. Fuel.

[126] Biela M., Rimarčík J., Senajová E., Kleinová A., Klein E. (2020). Antioxidant action of deprotonated flavonoids: Thermodynamics of sequential proton-loss electron-transfer. Phytochemistry.

[127] Wen L., Shi D., Zhou T., Tu J., He M., Jiang Y., Yang B. (2020). Identification of two novel prenylated flavonoids in mulberry leaf and their bioactivities. Food Chem..

[128] Yang X., Jiang Y., Yang J., He J., Sun J., Chen F., Zhang M., Yang B. (2015). Prenylated flavonoids, promising nutraceuticals with impressive biological activities. Trends Food Sci. Technol..

[129] Mvondo M.A., Njamen D., Kretzschmar G., Imma Bader M., Tanee Fomum S., Wandji J., Vollmer G. (2015). Alpinumisoflavone and abyssinone v 4′-methylether derived from Erythrina lysistemon (Fabaceae) promote HDL-cholesterol synthesis and prevent cholesterol gallstone formation in ovariectomized rats. J. Pharm. Pharmacol..

[130] Njamen D., Djiogue S., Zingue S., Mvondo M.A., Nkeh-Chungag B.N. (2013). In vivo and in vitro estrogenic activity of extracts from Erythrina poeppigiana (Fabaceae). J. Complement. Integr. Med..

[131] Kushwaha P.P., Prajapati S.K., Pothabathula S.V., Singh A.K., Shuaib M., Joshi K., Kumar S. (2020). Prenylated Flavonoids as a Promising Drug Discovery Candidate.

[132] Cui L., Lee H.S., Ndinteh D.T., Mbafor J.T., Kim Y.H., Le T.V.T., Nguyen P.H., Oh W.K. (2010). New prenylated flavanones from erythrina abyssinica with protein tyrosine phosphatase 1B (PTP1B) inhibitory activity. Planta Med..

[133] Khaomek P., Ichino C., Ishiyama A., Sekiguchi H., Namatame M., Ruangrungsi N., Saifah E., Kiyohara H., Otoguro K., Omura S. (2008). In vitro antimalarial activity of prenylated flavonoids from Erythrina fusca. J. Nat. Med..

[134] Ombito J.O., Majinda R.R.T., Masesane I.B., Bojase G., Schüffler A., Opatz T. (2018). Prenylated isoflavones from the stem bark of Erythrina sacleuxii. Phytochem. Lett..

[135] Passreiter C.M., Suckow-Schnitker A.K., Kulawik A., Addae-Kyereme J., Wright C.W., Wätjen W. (2015). Prenylated flavanone derivatives isolated from Erythrina addisoniae are potent inducers of apoptotic cell death. Phytochemistry.

[136] Togola A., Hedding B., Theis A., Wangensteen H., Rise F., Paulsen B.S., Diallo D., Malterud K.E. (2009). 15-Lipoxygenase Inhibitory Effects of Prenylated Flavonoids From Erythrina Senegalensis. Planta Med..

[137] Tuenter E., Zarev Y., Matheeussen A., Elgorashi E., Pieters L., Foubert K. (2019). Antiplasmodial prenylated flavonoids from stem bark of Erythrina latissima. Phytochem. Lett..

